# Ten Years on

**Published:** 1987-08

**Authors:** Rebecca B. Dunn, Mary E. McGraw

**Affiliations:** Senior Registrar in Geriatric Medicine, Southampton General Hospital; Consultant Paediatrician, Southmead Hospital


### Ten Years On

**Rebecca B. Dunn**
Senior Registrar in Geriatric Medicine, Southampton General Hospital
**Mary E. McGraw**
Consultant Paediatrician, Southmead Hospital

The careers of doctors qualifying in the United Kingdom over the decade 1974 to 1983 are being studied by a group now based in Oxford (1). As part of the study, the doctors who graduated from Bristol in 1976 were surveyed about career preferences during their pre-registration year (2), but unlike those qualifying in 1974, 1977, 1980 and 1983 they are not being followed to assess career progress. However as members of that year we were interested to know the careers that were being pursued and so while organising a ten year re-union which was held in August 1986, we collected data on our fellow graduates and have compared current careers with the preferences expressed almost a decade earlier.

Information was obtained on 100 of the 118 doctors who graduated from Bristol University Medical School in 1976. Ninety seven percent (58 men and 39 women) were practising medicine, 66% full-time and of those working part-time (1 man and 30 women), all were married with children. Forty nine percent were GPs and of the 9 consultants there were 3 pathologists (2 women), 2 anaesthetists, 1 geriatrician, 1 psychiatrist, 1 female paediatrician and 1 ophthalmologist. Seven of the 9 doctors who had passed finals with honours were in hospital practice, 2 as consultants and 5 as senior registrars. Eight of the 12 who had been awarded distinctions at finals were working in their distinction subject or a related specialty.

**Table**

**Careers predicted by Bristol University Medical graduates in 1976 (Parkhouse and Palmer) and actual careers in 1986 (figures are percentages of respondents and those in brackets refer to women)**

| | *1976* | *1986* |
|---|---|---|
| General practice | 41 | 49 (19) |
| Medicine | 21 | 6 (1) |
| Paediatrics | 7 | 5 (4) |
| Surgery | 13 | 10 (3) |
| Obstetrics & gynaecology | 2 | 2 (2) |
| Psychiatry | 3 | 9 (5) |
| Community medicine | 1 | 0 (0) |
| Pathology | 1 | 7 (5) |
| Anaesthetics | 5 | 6 (0) |
| Radiology/radiotherapy | 0 | 3 (0) |
| No prediction | 6 | — |
| Left medicine | — | 3 |

The Table shows that in 1976 21% predicted that they would be in a medical specialty but in 1986 only 6% were (3 in general medicine with a special interest, 2 in geriatric medicine and 1 in rheumatology). Of the 10% who had become surgeons, half, including the 3 women, were ophthalmologists while 4 were in general surgery and one was doing orthopaedics. Interestingly 9% were doing psychiatry although only 3% gave it as their first choice in 1976. Likewise more were doing pathology (7%) than had thought they would 10 years before (1%). In 1976 nobody chose radiology or radiotherapy but in 1986 3% were in this category. There were no community physicians.

Hutt, Parsons and Pearson (3) found that the most common reasons for doctors to change specialty were related to career prospects and job competition. In 1977 the DHSS notified doctors working in the NHS that career prospects in some specialties e.g. general practice, psychiatry, community medicine and some pathology subspecialties were good, whereas in others e.g. the surgical specialties and most medical specialties they were not and competition for posts would be strong for some years to come (4). Although we can only speculate as to why members of our year changed specialty such information is likely to have influenced some of us. Regular working hours and the availability of part-time posts were found to be important factors in the career decisions of women doctors (3). If these were reasons why several women in our year chose pathology, it is perhaps surprising that others did not opt for anaesthetics, radiology or community medicine. Clearly other factors were involved.

The study by Hutt and colleagues (3) showed that although most doctors chose their specialty within 10 years of qualifying, 9% (19% of women) decided later. Therefore we anticipate further changes in careers within our year and that more of the women will work full-time as their children grow up. We hope to document these changes 20 or 25 years on.

**REFERENCES**

1. ELLIN, D. J., PARKHOUSE, H. F., and PARKHOUSE, J. (1986) Career preferences of doctors qualifying in the United Kingdom in 1983. *Health Trends* **18**, 56–63.
2. PARKHOUSE, J. and PALMER, M. K. (1979) Career preferences of doctors qualifying in Britain in 1976. *Health Trends* **11**, 4–6.
3. HUTT, R., PARSONS, D. and PEARSON, R. (1980) The timing of and reasons for doctors' career decisions. *Health Trends* **13**, 17–20.
4. Medical Manpower Division, DHSS (1977) Medical Staffing and prospects in the NHS in England and Wales, 1976. *Health Trends* **9**, 45–47.

